# Expansion of *Capsicum annum* fruit is linked to dynamic tissue-specific differential expression of miRNA and siRNA profiles

**DOI:** 10.1371/journal.pone.0200207

**Published:** 2018-07-25

**Authors:** Dénes Taller, Jeannette Bálint, Péter Gyula, Tibor Nagy, Endre Barta, Ivett Baksa, György Szittya, János Taller, Zoltán Havelda

**Affiliations:** 1 National Agricultural Research and Innovation Centre, Agricultural Biotechnology Institute, Gödöllő, Hungary; 2 Department of Plant Science and Biotechnology, Georgikon Faculty, University of Pannonia, Keszthely, Hungary; Dokuz Eylul Universitesi, TURKEY

## Abstract

Small regulatory RNAs, such as microRNAs (miRNAs) and small interfering RNAs (siRNAs) have emerged as important transcriptional and post-transcriptional regulators controlling a wide variety of physiological processes including fruit development. Data are, however, limited for their potential roles in developmental processes determining economically important traits of crops. The current study aimed to discover and characterize differentially expressed miRNAs and siRNAs in sweet pepper (*Capsicum annuum*) during fruit expansion. High-throughput sequencing was employed to determine the small regulatory RNA expression profiles in various fruit tissues, such as placenta, seed, and flesh at 28 and 40 days after anthesis. Comparative differential expression analyses of conserved, already described and our newly predicted pepper-specific miRNAs revealed that fruit expansion is accompanied by an increasing level of miRNA-mediated regulation of gene expression. Accordingly, ARGONAUTE1 protein, the primary executor of miRNA-mediated regulation, continuously accumulated to an extremely high level in the flesh. We also identified numerous pepper-specific, heterochromatin-associated 24-nt siRNAs (hetsiRNAs) which were extremely abundant in the seeds, as well as 21-nt and 24-nt phased siRNAs (phasiRNAs) that were expressed mainly in the placenta and the seeds. This work provides comprehensive tissue-specific miRNA and siRNA expression landscape for a developing pepper fruit. We identified several novel, abundantly expressing tissue- and pepper-specific small regulatory RNA species. Our data show that fruit expansion is associated with extensive changes in sRNA abundance, raising the possibility that manipulation of sRNA pathways may be employed to improve the quality and quantity of the pepper fruit.

## Introduction

The fruit, this highly specialized complex organ, is the presumed crucial factor of the reproduction and dispersal success of angiosperm plants during evolution. Various types of tissues compose the complex structure of the fruit requiring sophisticated, multi-layered developmental processes during fruit formation. Understanding the molecular bases of fruit development is very important from a practical point of view as well since different types of fruits provide essential nutrients and minerals for healthy and balanced diet. Eukaryotic organisms, including plants, employ several RNA interference (RNAi) pathways. Some of these pathways work through small RNAs (sRNAs), 20 to 24 nucleotide in length, which mediate the negative regulation of their target RNAs in a sequence-specific manner. In plants, RNAi pathways play indispensable roles in development and biotic and abiotic stress responses. Distinct RNAi pathways generate different classes of small regulatory RNAs such as microRNAs (miRNAs) and small interfering RNAs (siRNAs) [1–3]. MiRNAs are crucial components in the fine regulation of gene expression during development. They modulate gene expression by regulating the degradation or translational repression of their target mRNAs [4]. Fully functional mature miRNAs are generated from their precursors by some phylogenetically conserved protein families [2] in the following manner: the nuclear genome-coded miRNA genes (*MIR*) are transcribed by RNA polymerase II (Pol II) to produce primary miRNAs (pri-miRNAs) possessing a specific and stable hairpin-like secondary stem-loop structure. In the nucleus, an RNase III enzyme, DICER-LIKE1 (DCL1) cleaves the hairpin out of the pri-miRNA resulting in the production of 80–300-nt-long miRNA precursors (pre-miRNAs). In plants, the same DCL1 enzyme then cleaves the precursor at sites determined by structural features to generate small double-stranded miRNA intermediates, the miRNA:miRNA* duplexes. These duplexes are 2’-O-methylated at their 3’ termini by the HUA ENHANCER 1 (HEN1) methyltransferase and then transported to the cytoplasm where they are loaded into the RNA-induced silencing complex (RISC). This complex displaces one of the strands (miRNA*) while the mature strand (miRNA) remains bound. The miRNA-containing RISC mediates sequence-specific repression via mRNA-cleavage or translational repression. The main executors of RISCs are the ARGONAUTE (AGO) proteins [5]. The *Arabidopsis thaliana* genome encodes ten AGO proteins which are specialized for various RNAi pathways but often display functional redundancies. AGO1 is the central component of the RISC playing a dominant role in miRNA-mediated regulation, and its expression is regulated by feedback mechanism-mediated by miR168 [6]. The generation of siRNAs is different from that of miRNAs because it is dependent on an RNA-dependent RNA polymerase (RDRP) [3]. The perfect double-stranded (ds)RNA intermediates generated by specific RDRPs can be recognized and cleaved by Dicer-like enzymes other than DCL1 to produce various classes of siRNAs [3]. HetsiRNAs are mainly 24-nt in length and generated by DCL3 via processing of Pol IV/RDR2-dependent dsRNA species originating from intergenic or repetitive regions of the genome. These 24-nt-long siRNAs are loaded into the RNA-induced transcriptional silencing (RITS) complex, which is functionally different from the RISC. They recognize Pol V-dependent short transcripts emerging from repetitive and intergenic regions and recruit chromatin-modifying complexes. HetsiRNAs play fundamental roles in maintaining genome integrity, by initiating *de novo* DNA methylation on transposable and repetitive elements mainly during reproductive transitions (meiosis, gametogenesis, and embryogenesis), especially in plants with larger, repetitive genomes. Phased (pha)siRNAs, however, are derived from Pol II-dependent mRNAs which are cleaved by a special class of miRNAs/siRNAs of 22-nt length [7]. This cleavage renders one of the cleavage products to be a substrate of RDR6/SUPPRESSOR OF GENE SILENCING 3 (SGS3). The resulting dsRNA is then cleaved by DCL4 endonuclease following a distinctive, regular (phased) pattern starting from the cleavage site. The phasiRNAs are preferentially loaded into a distinctive RISC containing AGO5. A subset of phasiRNAs, the trans-acting siRNAs (tasiRNAs) act mainly at the post-transcriptional level by negatively regulating their target transcripts including gene families which encode disease resistance proteins or transcription factors [8].

The continuously increasing number of experimental studies demonstrate that specific sRNAs regulate plant tissue and organ differentiation. Scientific experiments proved the importance of miRNAs and tasiRNAs in many aspects of plant development, including leaf, shoot, flower and root development, phase-change, auxin signaling and response to environmental/biotic stresses [9–12]. In addition to the well-known evolutionarily conserved miRNA families, there are non-conserved, lineage-specific miRNAs that are only present in closely related species [13, 14]. The identification of new miRNAs and the genome-wide, tissue-specific analyses of miRNA expression can help to specify miRNA-regulated developmental processes substantially affecting the quality of economically important traits of crop plants [15]. The role of miRNAs in fruit development has been extensively investigated by Next Generation Sequencing (NGS) technology [16–19]. Several studies analyzed the expression of miRNAs at a genome-wide level during fruit development in tomato, a *Solanaceae* model plant [20–24]. Fruit development in another *Solanaceae* species, the pepper (*Capsicum annuum*), is less extensively investigated. Conserved and potentially new, species-specific miRNAs have been described as a result of an investigation of different pepper organs including the developing fruit as a whole [25–28]. However, since the activity of miRNAs is spatiotemporally highly regulated [29–31], it is inevitable to study the expression pattern of miRNAs in a tissue-specific manner during the fruit development. Moreover, little is known about the contribution of sRNA-mediated regulation to the fleshy fruit development at the phase of cell expansion where the economically valuable fruit volume is often to reach a two magnitude fold increase.

To get high-resolution spatiotemporal information about dynamic sRNA expression pattern associated with the expansion phase of the pepper fruit development, we dissected the fruit. We collected flesh, seed, and placenta samples at 28 and 40 days after anthesis (DAA), and quantified their small RNA populations by high-throughput sequencing. Using the gained datasets, we generated the comparative tissue-specific differential expression profiles of the conserved, the already described, and the newly predicted pepper-specific miRNAs. We found that differential expression activity of miRNAs are intriguingly high at 40 DAA and especially the expansion of flesh is associated with profound changes in the miRNA expression profile. The pronounced role of miRNA-mediated regulation in flesh development was also reflected by the high level of AGO1 protein present in flesh samples. Moreover, the analyses of non-miRNA-like sRNAs revealed an ample of pepper-specific hetsiRNAs and phasiRNAs playing a role mainly in the development of the seed and the placenta. As a summary, our work demonstrates that the cell expansion phase of the economically important sweet pepper is associated with extensive changes in sRNA abundance implying that breeding technologies capable of modifying the expression of key sRNA regulators can be potent tools to enhance the economic value of pepper.

## Materials and methods

### Plant material

We used a Hungarian sweet cultivar of *Capsicum annuum* called Fehérözön for the experiments that we obtained from ZKI-Vetőmag Kft. (H-6000 Kecskemét, Mészöly Gyula u. 6). We germinated the seeds on plates between wet filter papers in a growth chamber (stable 21°C, in darkness). We planted the seedlings into pots and grow them in a light room under long day (16 h light/8 h dark) conditions. We collected the fruits at 28 and 40 days after anthesis (DAA) and dissected to separate the seed, the placenta and the flesh tissues.

### RNA extraction and small RNA library construction

We isolated total RNA using Trizolate Reagent (UD Genomed Ltd., Debrecen, Hungary) from the different tissues. We determined the RNA concentration and 260/280 ratio with NanoDrop spectrophotometer (Thermo Scientific, Wilmington, DE) and verified the RNA integrity by agarose gel electrophoresis. We separated 30 μg of total RNA per sample on an 8% denaturing polyacrylamide/urea gel along with RNA size markers, and then isolated the sRNA range. The pellets were disolved in 20 μl RNase-free ultrapure water, of which we used 2.5 μl for library preparation. We constructed the sRNA libraries using the Truseq Small RNA Sample Prep Kit (Illumina, CA, US), according to the manufacturer's instructions. We submitted the pools of eight PCR-amplified, gel-purified and bar-coded cDNAs to UDGenomed Ltd. (Debrecen, Hungary) for sequencing on Illumina HiSeq 2000 platform.

### Bioinformatic analysis

We processed the the raw sequences using cutadapt 1.9.1. [32]. We performed a quality check before and after processing with FastQC 0.11.5 [33]. For miRNA analysis, we aligned the clean reads to the reference genome of *Capsicum annuum* cv. CM334 (version 1.55). We used MirCat [34] and miRDeep-P [35] in parallel to find new miRNAs; then we combined and filtered the two datasets by custom Python scripts. We used three subsets: miRNAs from the papers [25, 26] describing pepper genome and miRNA sequencing, miRNAs from MirProf results and miRNAs from mirCat and miRDeep-P predictions. We determined the raw expression values by Patman [36], and we used custom Python scripts to create a raw, and also a normalized abundance matrix (Tables A-C in S2 File). We performed the differential expression analysis with DESeq2 [37] on the raw abundance matrix. We got the basemean values and the shrunk fold-changes (in log2 form) from DESeq2. We considered those miRNAs differentially expressed, whose basemean value was at least 10 and shrunk fold-change was higher than 6. For the visualization of the results of the differential expression analysis, we created heat maps and MA-plots. We carried out the clustering of miRNAs into families with cd-hit EST [38] by setting the sequence identity cut-off to 0.8. For multiple alignments of the sequences of miRNA families, we used Clustal Omega, Color Align Conservation [39] and WebLogo [40] applications.

We identified the sRNA producing genomic clusters with ShortStack 3.4 [41]. During the alignment, we allowed one mismatch and a maximum of 1000 possible placements for multi-mappable reads. To allow reporting the predominant RNA size in all loci, we set the parameters called dicermin and dicermax to 18 and 35, respectively. We kept all the other parameters at the default value. To identify differentially expressed loci between tissues and developmental stages, we applied DESeq2 with the filtering parameters used for miRNAs. We carried out the annotation of the loci with Patman using the Zunla RNA and TE sequences.

### Western blot analysis

We homogenized tissue samples in an ice-cold mortar in 400 μl 2× Laemli buffer. Protein samples were resolved on 8% SDS-polyacrylamide gel and blotted to PVDF Blotting Membrane (Amersham^TM^ Hybond^TM^) with Trans-Blot Turbo (BIO-RAD) and subjected to Western blot analysis. We blocked the membrane with 5% non-fat milk powder in PBS containing Tween 20 (PBST) for 1 hour. We probed the membrane with anti-*Arabidopsis thaliana* AGO1 (Agrisera) in PBST containing 5% non-fat milk powder at room temperature for three hours. After washing the membrane in PBST, we added the secondary peroxidase-conjugated goat anti-rabbit IgG (H&L). The signals were visualized by chemiluminescence (Clarity^TM^ Western ECL substrate; BIO-RAD) according to the manufacturer’s instructions. We stained the membrane with Ponceau reagent to check even protein loading.

### Small RNA Northern blot analysis

For the sRNA analysis, we separated 5 μg of total RNA on denaturing 12% polyacrylamide gel containing 8 M urea and transferred to Hybond^TM^-NX membrane (Amersham) with Fast Blot (Biometra). RNA was chemically crosslinked to the membrane (2008 Pall and Hamilton). We hybridized the membranes with radioactively labeled LNA or DNA oligonucleotide probes to detect sRNAs [42]. We end-labeled 10 pmol of each oligonucleotide probe with [γ-^32^P]ATP and T4 polynucleotide kinase. The hybridization was carried out at 37°C (DNA) or 50°C (LNA) depending on the type of oligonucleotides. We washed the membrane for 5 min two times with washing solution containing 0.1% SDS and 2× SSC at the temperature of the hybridization. We listed the used oligonucleotide probes in Table rr in S2 File.

## Results and discussion

### Small RNA sequencing and data pre-processing

**Fig 1:**
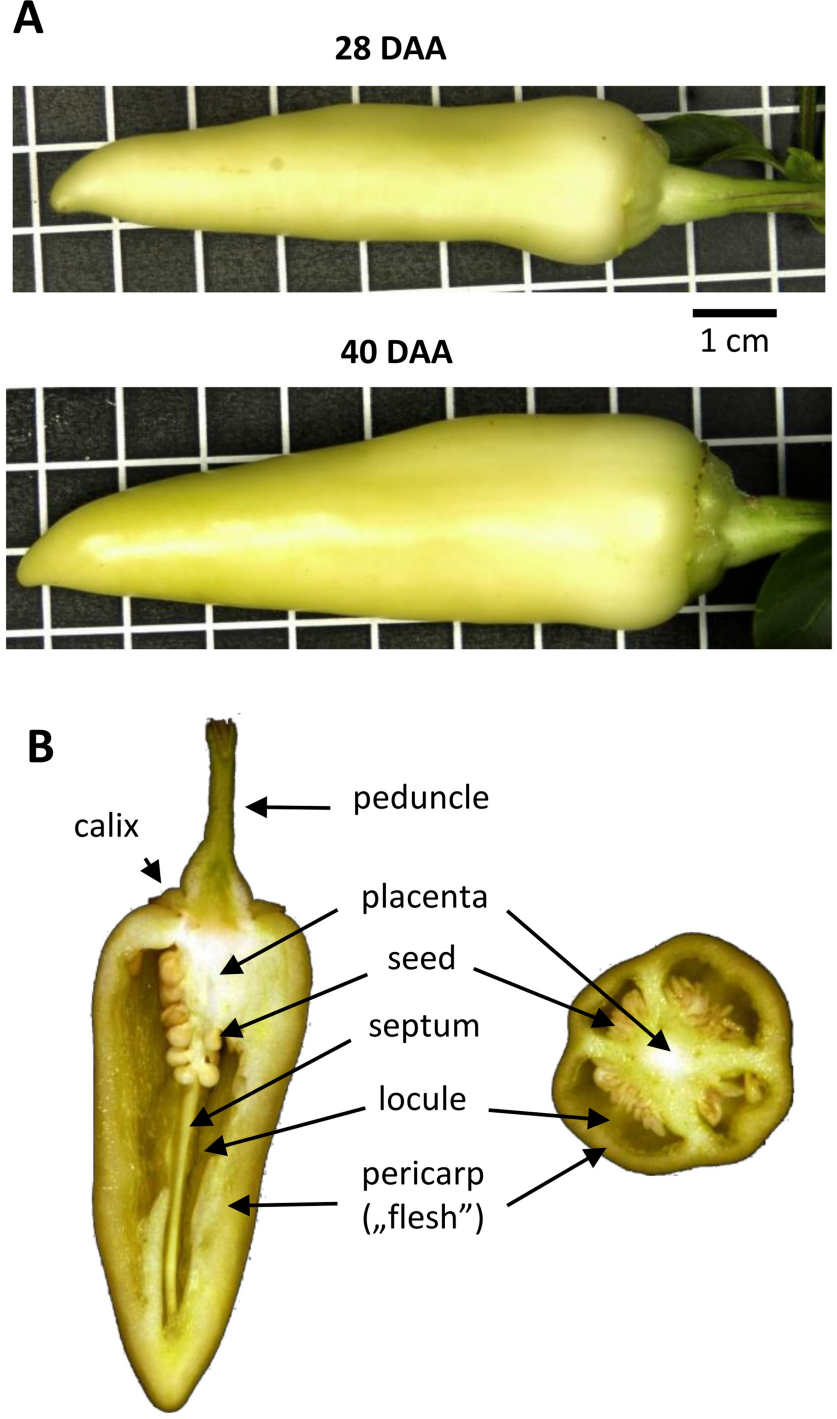
Developmental stages of the sweet pepper cv. Fehérözön investigated in this study. (A) The two selected fruit developmental stages at 28 and 40 days after anthesis (DAA). Grid = 1 cm. (B) Vertical and horizontal sections of expanding fruit showing the composing tissues as indicated. The seeds, the placenta, and the flesh were isolated and used for RNA extractions separately.

In this study, we used high-throughput sequencing method to analyze the tissue and development-specific expression of sRNAs in the fruit development of sweet pepper (*Capsicum annuum* cv. Fehérözön). We collected samples at 28 and 40 DAA (Fig 1A). At these stages, the phase of rapid cell division is completed, and the development is already in the cell expansion phase. Since cell expansion accounts for the largest increase in fruit volume, the regulation of these developmental stages are extremely important from an economic point of view. To obtain tissue-specific sRNA expression profiles, we dissected the pepper fruit. Seed, placenta and flesh tissues were collected separately (Fig 1B and Figure A in S1 File) and processed for sRNA sequencing in two independent biological repeats.

A total of more than 135 million reads were generated, in the range of 4.4–17.5 million reads per individual samples (Table 1). After removing adaptor sequences and filtering out low-quality tags, a total of 131.19 million clean reads were obtained, which were 16–28-nt in length. Next, we removed low abundance (< 2) reads, and those that aligned to known non-coding RNAs (rRNA, snRNA, snoRNA, tRNA), which resulted in 74.8 million filtered reads. More than 85% of these filtered reads matched the genome (version 1.55) of pepper cultivar CM334 [27] perfectly. The most critical filtering step was the removal of sequences with low abundance. The small numbers of sequences with an invalid length, low-quality tags, or low complexity indicate that the quality of our libraries was appropriate.

**Table 1:** Summary of the read numbers along the filtering steps in the small RNA libraries.

		Raw	Trimmed	Filter1	Filter2	Aligned
28 DAA	Seed 1	17.51	17.33	11.29	10.72	9.00
	Seed 2	12.18	11.79	8.43	7.90	6.82
	Placenta 1	6.18	5.44	2.78	2.65	2.30
	Placenta 2	16.22	16.08	8.54	8.39	7.39
	Flesh 1	11.69	10.42	9.56	9.03	6.49
	Flesh 2	4.41	4.02	2.42	2.26	1.99
40 DAA	Seed 1	9.27	9.15	4.04	3.79	3.37
	Seed 2	12.62	12.43	6.57	6.21	5.61
	Placenta 1	12.67	12.48	6.34	6.11	5.23
	Placenta2	15.11	14.99	8.08	7.77	6.70
	Flesh 1	8.91	8.78	5.23	4.84	4.26
	Flesh 2	8.35	8.29	5.32	5.15	4.65
	Total	135.13	131.19	78.60	74.82	63.81

Filter 1: removal of reads with low abundance (1–2). Filter 2: removal of tRNA, rRNA, snRNA and snoRNA and low complexity sequences. Aligned: reads that matched the pepper genome. The numbers denote millions of reads.

### Size distribution of the small RNA sequences

**Fig 2:**
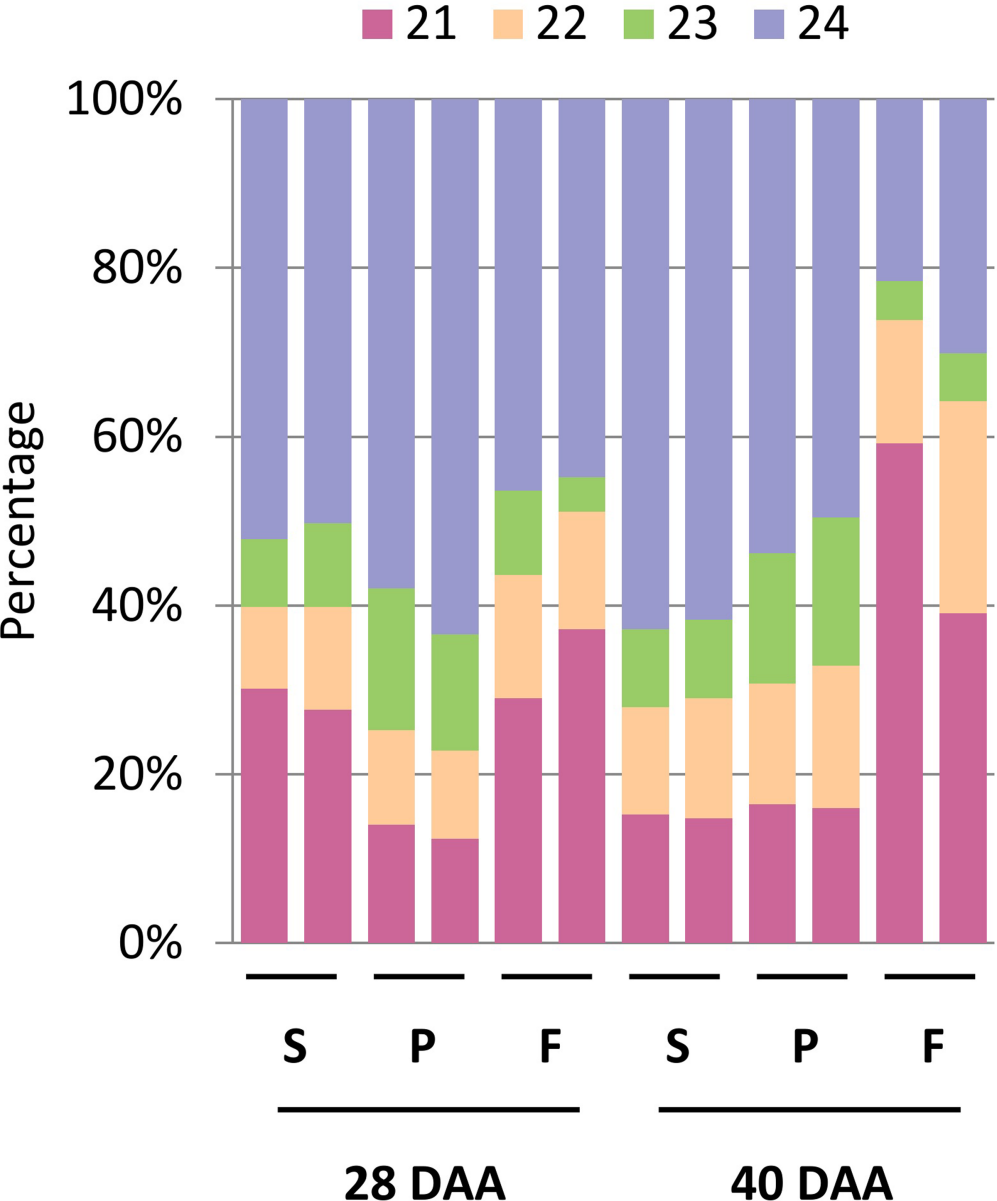
Size distribution of small RNAs in the sequencing libraries. The diagram shows the small RNA size composition of each library. Biological replicates are displayed next to each other. Numbers at the top indicate the length of sRNAs.

The size distribution of all the filtered sequences also confirms the high-quality of sRNA sequencing data since more than 94% of the filtered sequences fall into the 21–24-nt size range, while the 16–20-nt and the 25–28-nt range represent only 4.8% and 1%, respectively (Fig 2).

The 46.73% of the total sRNAs were 24-nt-long while 24.05% were 21-nt-long. The 22 and 23-nt-long sRNAs were the less abundant classes, 14%, and 10%, respectively (Fig 2). The analysis of the individual samples revealed that the 24-nt-long sRNA species was the dominant size class in the majority of the samples [13, 43]. However, we observed an increased ratio of 21-nt-long sRNAs in the flesh at 28 DAA and even more prominently at 40 DAA (Fig 2) indicating that the proportion of different size classes of sRNAs can vary significantly during the pepper fruit development. To analyze this phenomenon, we calculated the 21/24-nt ratio of the different samples and observed differences between various tissue types and developmental stages. The 21/24 ratio of the placenta samples increased only moderately, while in developing seeds it decreased from 55% (seed 28 DAA) to about 25% (seed 40 DAA). In the flesh, however, it increased from 75% (flesh 28 DAA) to about 200% (flesh 40 DAA). We found the ratio of the 21-nt-long sRNAs increasing during the expansion of the fruit except for the seed. The dynamic changes in the relative accumulation of various sRNA species with the advance of time indicate that sRNAs may play a pivotal role during fruit expansion. Moreover, the high percentage of the 21-nt-long sRNA species in the flesh suggests that miRNAs, phasiRNAs, or other 21-nt-long sRNA molecules play a crucial role in the development of the economically most important tissue of the pepper fruit.

### Conserved miRNAs in the developing pepper fruit

Two approaches have been applied to identify known miRNAs in the pepper fruit-specific sRNA libraries. MirProf has been used to profile known miRNAs by allowing two mismatches. MirProf uses Patman to align reads against miRBase [44]. MiRBase currently does not contain any pepper miRNAs, so we aligned our reads to the recently published conserved pepper miRNAs (can-mir) using Patman with the same parameters [25, 27]. We discarded sRNA reads, which were not present at least in two libraries (213 different sequences remained). However, this dataset contains some 18-nt-long sequences, which we did not consider for further analysis. Our final set of conserved miRNAs contained 193 non-redundant sequences, from 38 conserved miRNA families (Table A in S2 File). The majority of these miRNAs are in the mirBase database with two mismatches: 143 in *Solanum tuberosum*, 132 in *Solanum lycopersicum*, and 110 in *Nicotiana tabacum*.

A recent comprehensive survey of plant miRNAs from 31 vascular plants species specified 82 known miRNA families that can be sorted into eight groups depending on their distribution across lineages and species [45]. The first group contains miRNA families that are widely present and highly expressed in all terrestrial species. In our samples, all of these miRNA families expressed at high level according to the RPM data (*miR156*: 249 RPM, *miR166*: 466210 RPM, *miR167*: 95 RPM, *miR168*: 4444 RPM), except for the *miR172* family, which expressed at a lower level (17 RPM). The group 2 miRNA families are represented in all taxonomic lineages but can be absent or present with low abundance in some species (*miR158*, *miR159*, *miR160*, *miR162*, *miR164*, *miR169*, *miR171*, *miR319*, *miR390*, *miR393*, *miR394*, *miR396*, *miR397*, *miR529*, *miR535* and *miR4414*). Most of the group 2 miRNA families were also present in our data sets except for the *miR158*, *miR529*, and *miR535* families. Seven families (*miR159*, *miR162*, *miR169*, *miR171*, *miR319*, *miR393*, *miR396*) were abundant (more than 75 RPM) at least in one library. Other six families (*miR160*, *miR164*, *miR390*, *miR394*, *miR397*, *miR4414*) were moderately abundant (around 5–50 RPM). *MiR158*, *miR529*, and *miR535* were absent from all the investigated pepper libraries. In a recent study investigating *Nicotiana benthamiana* sRNAs, *miR529* and *miR535* were absent from sRNA libraries [46]. One of the other six groups, group 8, was enriched in *Solanaceae* species, like *Petunia*, *Nicotiana tabacum*, *Solanum lycopersicum*, *Solanum tuberosum* and *Capsicum annuum*. From the ten miRNA families present in this group (*miR1919*, *miR4376*, *miR5300*, *miR5301*, *miR6022*, *miR6023*, *miR6024*, *miR6025*, *miR6026*, *miR6027*, *miR6149*), we identified 5 (*miR4376*, *miR5300*, *miR5301*, *miR6024*, *miR6027*) in our libraries. We checked the missing miRNA families in the libraries analyzed by Chavez and his colleagues, and some of them expressed weakly in their pepper samples, too. One example is *mir1919*, which is missing from all of their pepper libraries.

### Known pepper-specific miRNAs in the developing pepper fruit

Recent studies described new pepper-specific miRNAs [25, 27, 28]. Based on published pepper miRNA sequences, we prepared a merged list of known pepper-specific miRNA sequences. The latest paper on pepper fruit miRNAs [28] described four sequences (*Novel_miR265*, *Novel_miR218*, *Novel_miR23*, *Novel_miR279*) from our set of new miRNAs, so we transferred these miRNAs to the known pepper-specific group, with the IDs given in that paper. The other published two sets of new miRNA sequences have only two common elements: *can-miR-n010/can-miRC7-3p*.*1* and *can-miR-n003a/can-miRC13-5p*. We created a non-redundant list of 68 sequences and we found 50 of these sequences (including the two common ones) in our libraries (Table B in S2 File). We found more than 83% of the miRNAs described by Hwang et al. and 33% of the miRNAs described by Qin et al. [25, 27]. We did not detect some of the miRNAs, such as *can-mir-n05* and *can-mir-n027*, possibly because they are expressed in the root and the stem but not in the fruit. However, we detected some known miRNAs which appeared to be flower- or leaf-specific in the studies mentioned above (i.e., *can-mir-n019a-d* and *can-mir-n030*).

### New miRNAs in developing pepper fruit

**Fig 3:**
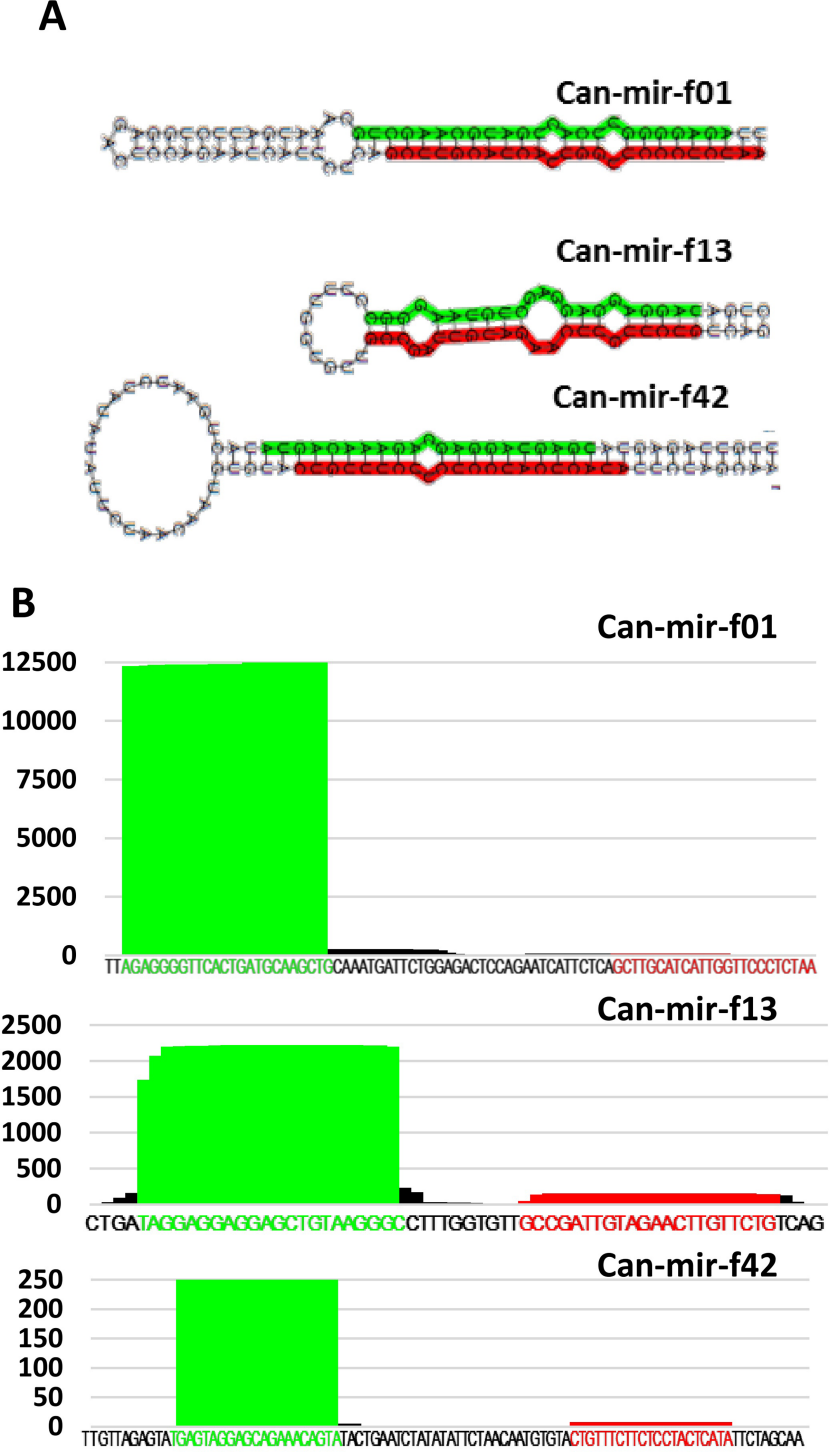
Novel miRNA representatives. **(**A) Read alignment diagram for the selected miRNA precursors. Mature strand is highlighted with green and star strand with red. (B) Predicted secondary structure of pre-miRNAs (Mfold). Mature miRNA and miRNA* sequences are highlighted.

To reveal still unknown miRNAs, we constructed a novel miRNA identification pipeline (Figure B in S1 File). 74.82 million filtered reads were used to identify new miRNAs by paralleled utilization of two computational plant miRNA prediction tools, miRDeep-P, and miRCat. Both of these tools map the reads to the genome and extract reference sequences by extending mapped reads in the flanking regions based on their ability to fold predicted miRNA precursor RNA stem-loop structures. These programs use the Vienna RNA package [47] to determine the secondary structure of the predicted precursors and apply a scoring system to rank the obtained potential miRNAs precursor structures. Although these programs use similar computational approaches, they can generate significantly different results. To enhance the reliability of our miRNA predictions, we used both programs to identify the potentially new pepper miRNAs and accepted only the overlapping set of different predictions. The next filtering steps were the elimination of candidate reads which did not reach the abundance level of 10 RPM and appeared at least in two sRNA libraries. Utilization of this method allowed us to describe 73 different, highly confident novel pepper miRNA sequences (Table C in S2 File). In all cases, we detected the miRNA* sequence in at least one of our libraries; also their predicted precursors have a stable secondary structure (Table D in S2 File). We were able to cluster some of the novel miRNAs into six miRNA families, but the majority of them are singletons (Figure C in S1 File). We also checked the similarity between our newly described and the previously described miRNAs and found that five (*can-mir-f11*, *can-mir-f14*, *can-mir-f27*, *can-mir-f46*, *can-mir-f57*) of our novel miRNAs were similar to previously described miRNAs [28]. These family members differ mainly in their sequence composition indicating that they probably derive from different precursors. We show the structures of some selected novel miRNA precursors in Fig 3.

### Validation of the miRNA expression profiles

**Fig 4:**
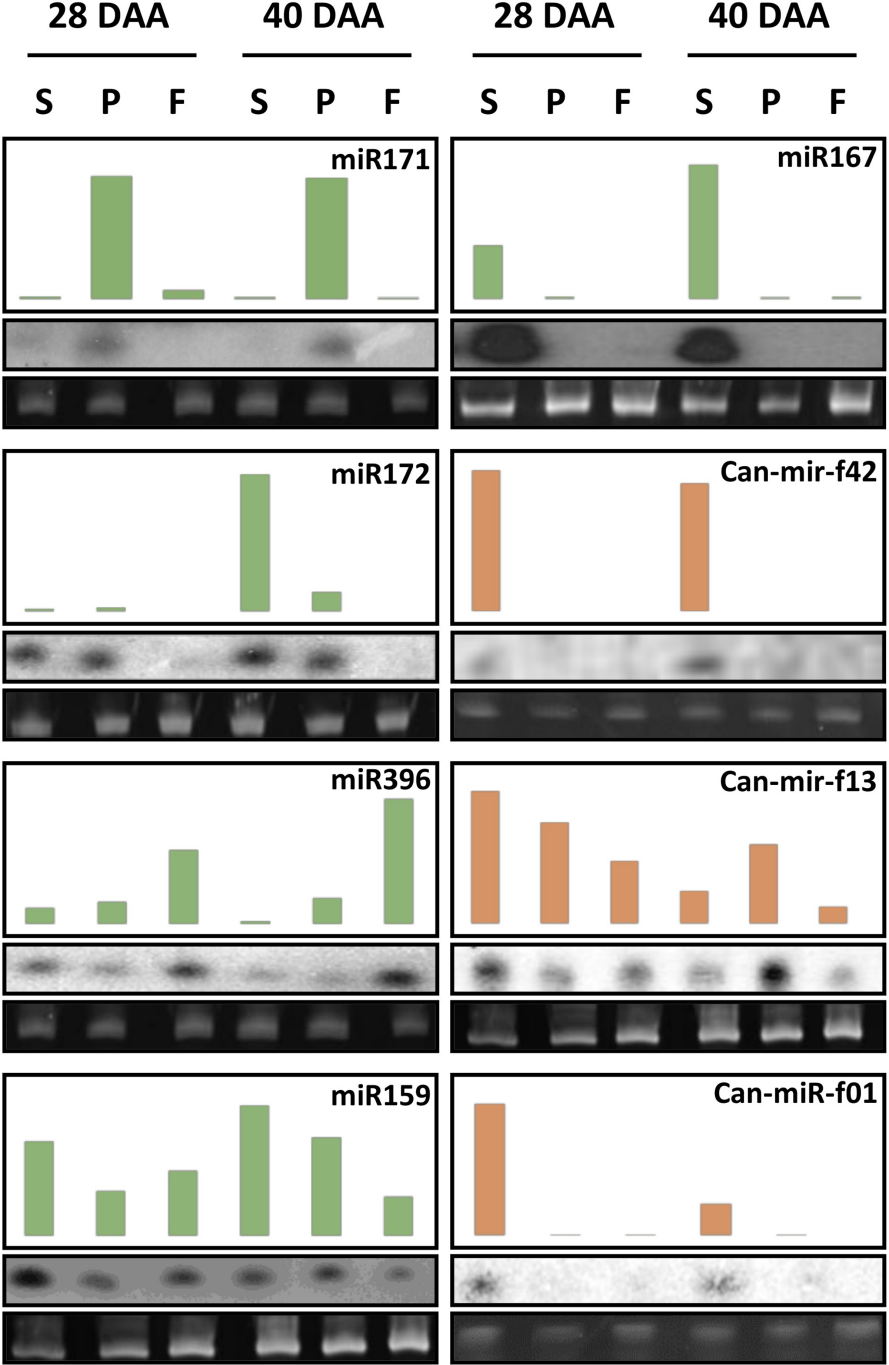
Small RNA Northern blot analyses of selected miRNAs. Expression of the conservative (green) and the newly predicted (red) miRNAs. The total RNA samples from the seed, the placenta and the flesh at 28 and 40 days after anthesis (DAA) were run on agarose gels, transferred to nylon membranes, and hybridized with probes detecting miRNAs as indicated. We show the NGS read counts (read/million) in the upper panels, the results of small RNA northern blots in the middle panels, and the rRNA levels as loading controls in the lower panels. We used the same membrane for the hybridization of *miR171*, *miR396*, and *miR159*, *can-mir-f13* specific probes.

We validated the results by sRNA Northern blot analysis. We used DNA, or LNA oligonucleotide probes to detect the expression patterns of selected conserved miRNAs (*miR171*, *miR172*, *miR396*, *miR159*, and *miR167*) (Fig 4, green bars). The result of the Northern analysis was in a good agreement with the sequencing data, although in some cases we observed discrepancies. This could be attributed to the fact that the hybridization-based technology measures the abundance of heterogeneous miRNA population depending on the parameters of the hybridization procedure. Next, we analyzed the expression patterns of selected new pepper-specific miRNAs (Fig 4, red bars). Similarly to the expression of the conserved miRNAs, the experimental analyses revealed highly tissue-specific expression of the novel miRNAs. Altogether, these data indicate that the gained sRNA sequencing data are suitable for differential expression analysis of miRNAs during the developmental process.

### Differential miRNA expression in the pepper fruit

**Fig 5:**
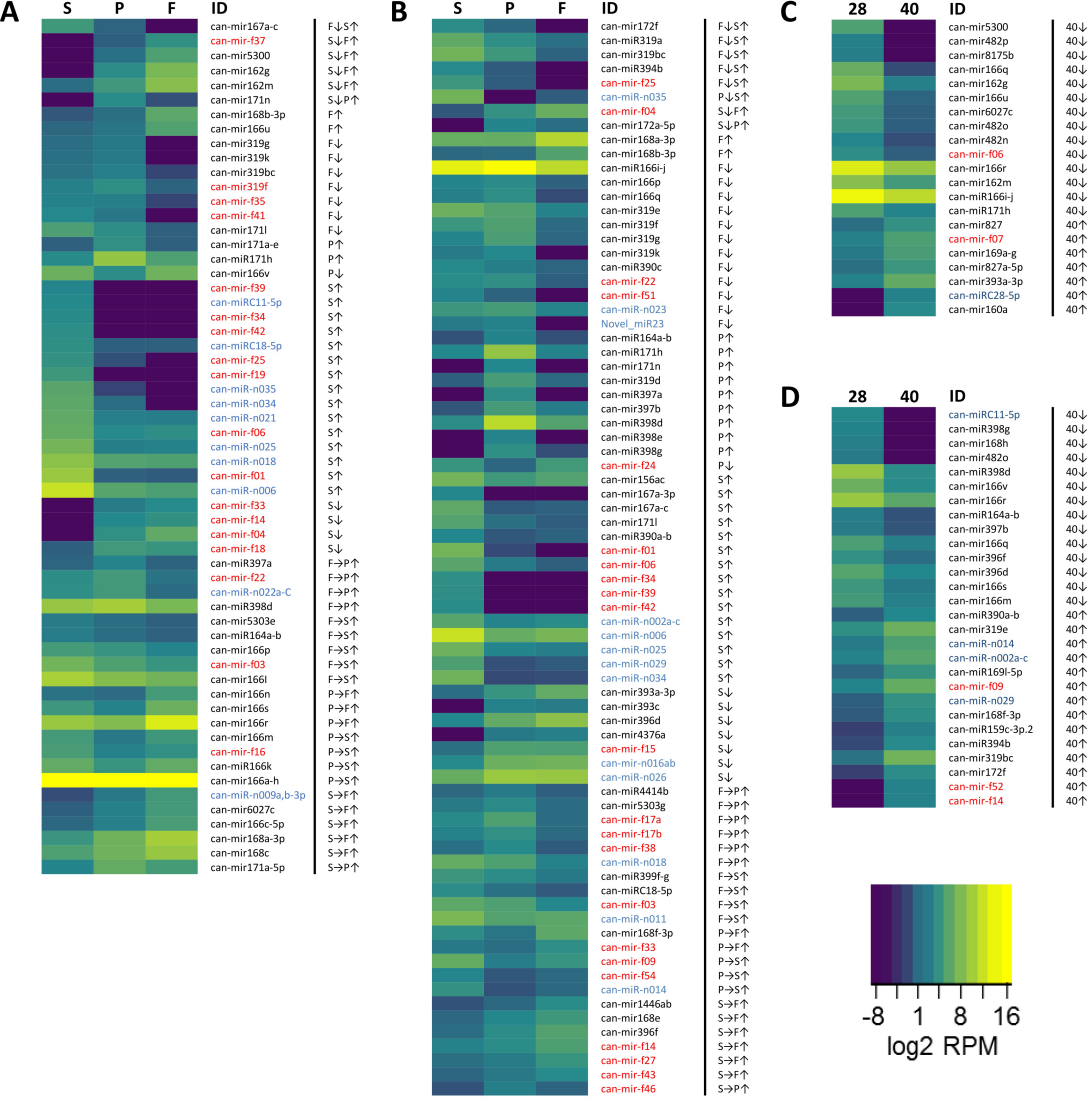
Heat map showing the expression levels of the differentially expressed miRNAs. The differential expression analysis between the seed (S), the placenta (P), and the flesh (F) samples at (A) 28 DAA and (B) 40 DAA was conducted using DESeq2. The results were filtered to keep only those miRNAs whose mean normalized expression level was at least 10, and the expression change was at least six-fold. We colored the conservative (black), known (blue) and novel (red) miRNAs. We found 21 differentially expressed miRNAs between different stages (28, 40) in the flesh (C), 28 miRNAs in the seed (D), and none in the placenta. Arrows represent up- (↑) and down-regulation (↓) compared to the two other tissues (for example S↑ means, the expression of the marked miRNA are upregulated in seed compared to both, placenta and flesh. We also listed those changes, which occurred only between two tissues. For example, we marked a miRNA up-regulation between the placenta and the seed like this: P→S↑.

To gain insights into the putative roles of the identified miRNAs, we conducted a differential expression analysis between the tissues and developmental stages using DESeq2. First, we determined the differentially expressed miRNAs between the tissues at 28 DAA (Fig 5).

We detected 20, potentially influential conserved miRNAs from nine families, four new, and one known pepper-specific differentially expressed miRNAs in the placenta relative to the flesh and the seed (Tables E-F, N-O, and W-X in S2 File). The comparison of the placenta and the seed samples revealed less conservative (13), but more known pepper-specific (9) and new (13) differentially expressed miRNAs. We detected an increased number of changes between the flesh and the seed samples, where 44 miRNAs displayed significantly altered expression (Tables G, P, and Y in S2 File). Altogether, the majority of the differentially expressed miRNAs were conservative, and they show both up and downregulation in all comparisons. At this stage, differential expression of known and new pepper-specific miRNAs mostly related to their altered expression levels in the seed since fewer changes were detected between the placenta and flesh samples. At 40 DAA, we observed extensive changes in expression profiles of all miRNA classes between every tissue (Fig 5, Figure E in S1 File, Tables H-J, Q-S, and Z-bb in S2 File). The majority of miRNAs with altered accumulation expressed differentially in more than one comparisons. We found miRNAs in all classes, which showed significantly different levels of expression in all tested tissues (*can-mir172a-5p*, *can-mir172f*, *can-miR319a*, *can-mir319bc*, *can-miR394b*, *can-miR-n035*, *can-mir-f04*, and *can-mir-f25*). The differentially expressed conservative miRNAs at this stage (40 different mature sequences) are mainly downregulated in comparison of placenta and flesh samples while in the other comparisons up-, and downregulation of miRNAs are mostly balanced. The known pepper specific miRNAs, similarly to the 28 DAA stage, are mostly upregulated specifically in the seed. The majority of the changes in the expression level of new miRNAs are also related to seed, but we identified expression differences between flesh and placenta samples as well.

We also compared the miRNA expression profiles between 28 and 40 DAA in the different tissues (Fig 5C and 5D, and Figure F in S1 File). We observed significant differences in the case of the seed and the flesh samples (Tables L-M, U-V, and dd-ee in S2 File). The majority of the differentially expressed sequences were conservative miRNAs, and only limited changes were found in the expression of pepper-specific known and novel miRNAs. The placenta, however, showed practically no changes in miRNA expression profiles (Tables K, T, and cc in S2 File). The fact that most of these changes affected mostly the conservative miRNAs suggests evolutionary conservation of the miRNA-mediated regulation of the fruit tissue development during the expansion phase. Altogether, these data suggest that the investigated developmental stages are associated with specialized miRNA expression patterns and that the miRNA-mediated regulation is prevalent during the expansion phase.

### Expression of AGO1 protein in the pepper fruit tissues

**Fig 6:**
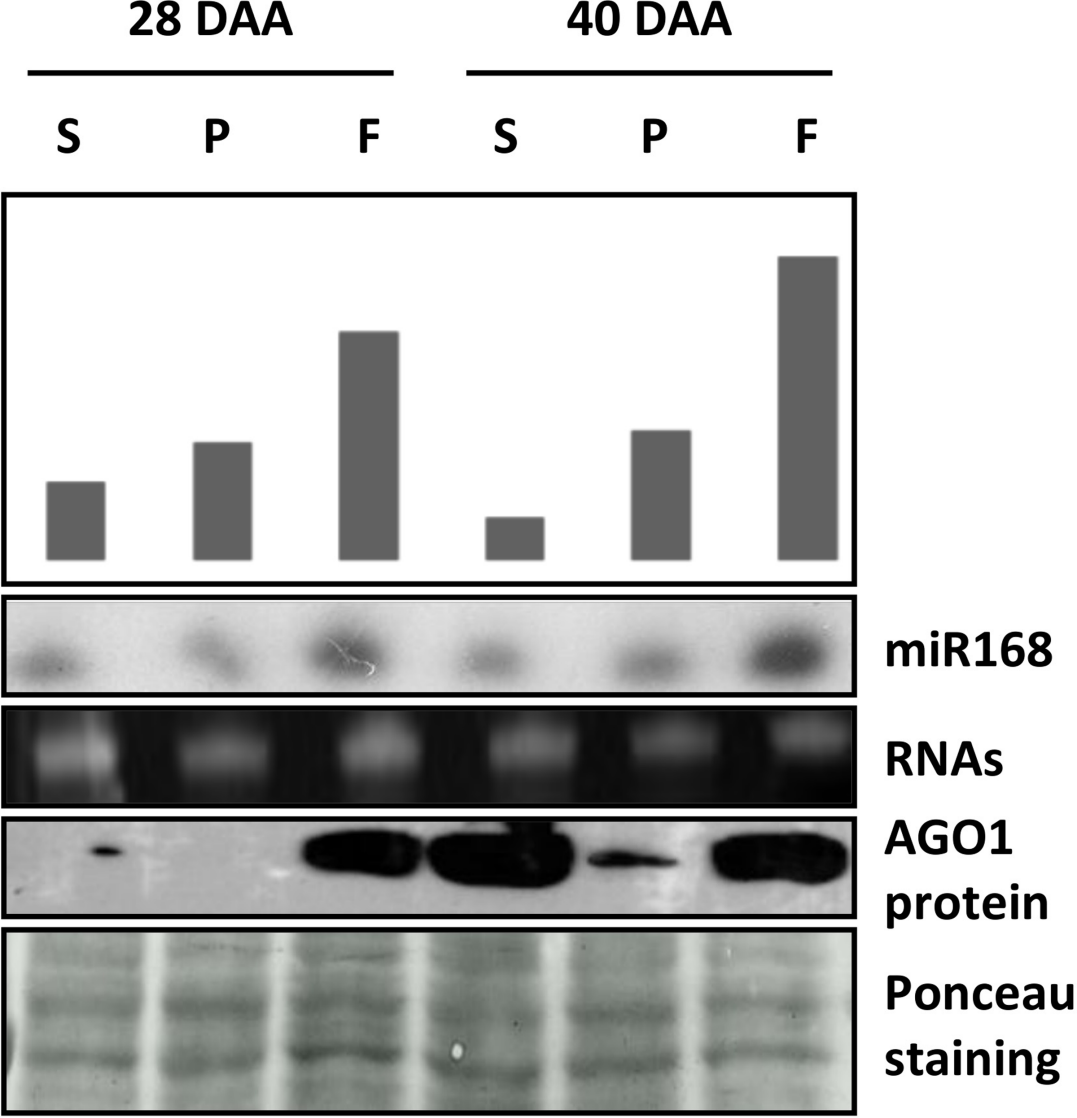
Accumulation of AGO1 protein and *miR168* in pepper fruit tissues. RNA and protein samples of seed, placenta, and flesh, representing developmental stages at 28 and 40 days after anthesis (DAA), were used for small RNA northern blot analyses and Western blotting. Upper panel is the schematic representation of miR168 NGS read counts (read/million). Middle panels show the results miR168 northern blots. EtBr stained rRNAs serve as loading control. Bottom panels show Western blot analyses of total protein extracts for AGO1 accumulation. Equal loading was verified by Ponceau staining of the membrane after western blotting.

We found that the pepper fruit expansion is associated with profound temporal changes in the miRNA expression profile which can indicate the central role of miRNA-mediated regulation in this developmental process. AGO1 is the most important AGO protein in the miRNA pathway which is responsible for the cleavage or translational inhibition of target mRNAs determined by the loaded miRNA. AGO1 homeostasis is controlled by the action of *miR168* on *AGO1* mRNA [6]. Next, we investigated the accumulation of AGO1 protein, as the key executor component of RNAi, postulating that the accumulation level of AGO1 reflects the activity RNAi in the particular tissue types. First, we analyzed the accumulation of the AGO1 key regulator *miR168* by sRNA Northern blot. We found, in agreement with the NGS data, that *miR168* expresses abundantly in general with the highest level in the flesh samples at both time points (Fig 6). Using protein extracts representing the same samples we analyzed the expression of AGO1 with western blot. At 28 DAA no significant AGO1 accumulation was detected in the seed and the placenta samples at the detection level of the used method while in the flesh sample AGO1 accumulated at very high level. At 40 DAA we experienced drastic and moderate AGO1 induction in the seed and the placenta samples and importantly a continuously high AGO1 expression in the flesh.

In accordance with the differential expression results, these data indicate that extensive miRNA expression changes are associated with the expansion of the pericarp. The importance of miRNAs in the expansion of the flesh is further supported by the observation that the proportions of the 21-nt-long sRNAs are highest in the flesh samples, especially at 40 DAA, where the 21/24-ratio becomes higher than one (Fig 2). In the placenta, the AGO1 level remains relatively low suggesting that temporal changes in the miRNA expressions are not required for the proper formation of this tissue. Indeed, the differential expression of the pattern of the placenta samples at the two investigated time points showed no changes (Figures D-F in S1 File). In the case of the seed, a very low AGO1 expression is detected at the early time point, however, in the later time point, the amount of the AGO1 protein drastically increased probably due to the increasing size of the embryo (Figure A in S1 File). We also found that the levels of *miR168* in the samples do not correlate with the amount of AGO1 protein suggesting a potentially unusual regulation. This phenomenon requires further investigation.

### Expression of 24-nt-long hetsiRNA clusters

The most abundant class of endogenous siRNAs are 24-nt-long and are predominantly produced from transposons and repeated sequence elements, especially heterochromatic regions [48]. Genome associated siRNAs are engaged in various nuclear processes such as developmental gene and transposon regulation, heterochromatin formation, and genome stability [49]. It has also been shown that 24-nt-long siRNAs play a crucial role in transgenerational epigenetic inheritance [50]. To analyze the expression pattern of the 24-nt siRNA-producing clusters during pepper fruit expansion, we investigated our datasets using ShortStack [41]. We identified 1,012,092 sRNA-producing clusters altogether. Most of the *de novo* identified siRNA clusters associated with the dominant sequence length of 24-nt (850,801; 84.1%). Of these clusters, 426,309 (42.1%) appeared to be associated with sequence repeats or mobile genetic elements. Others are localized to the promoters or gene bodies of protein-coding genes, probably affecting the methylation state of the DNA and consequently the transcriptional activity. We performed a differential expression analysis on the non-miRNA producing sRNA clusters using DESeq2 in the same way as in the case of miRNAs. Pairwise expression changes were graphically represented on MA-plots to visualize the distribution of expression values and differences (Figure G in S1 File). The associated information provided by ShortStack and DESeq2 for the significantly changed clusters are listed in Tables ff-nn in S2 File. We can observe a similar trend in changes between tissues and time points as in the case of miRNAs. Most importantly, there are only a small number of significantly changed clusters in the placenta samples between the two time points, a moderate number of changing clusters between the placenta and the flesh samples, whereas the greatest changes can be observed between the seed and other tissues. Also, it is noteworthy that the expression levels of most of the sRNA clusters are higher in the seed samples at 40 DAA than at 28 DAA. This can be explained by the growth and differentiation of the embryonic tissue in the seed (Figure A in S1 File), suggesting that the 24-nt-long hetsiRNA-producing clusters are more active in this tissue to prevent genome instability during embryonic development.

**Fig 7:**
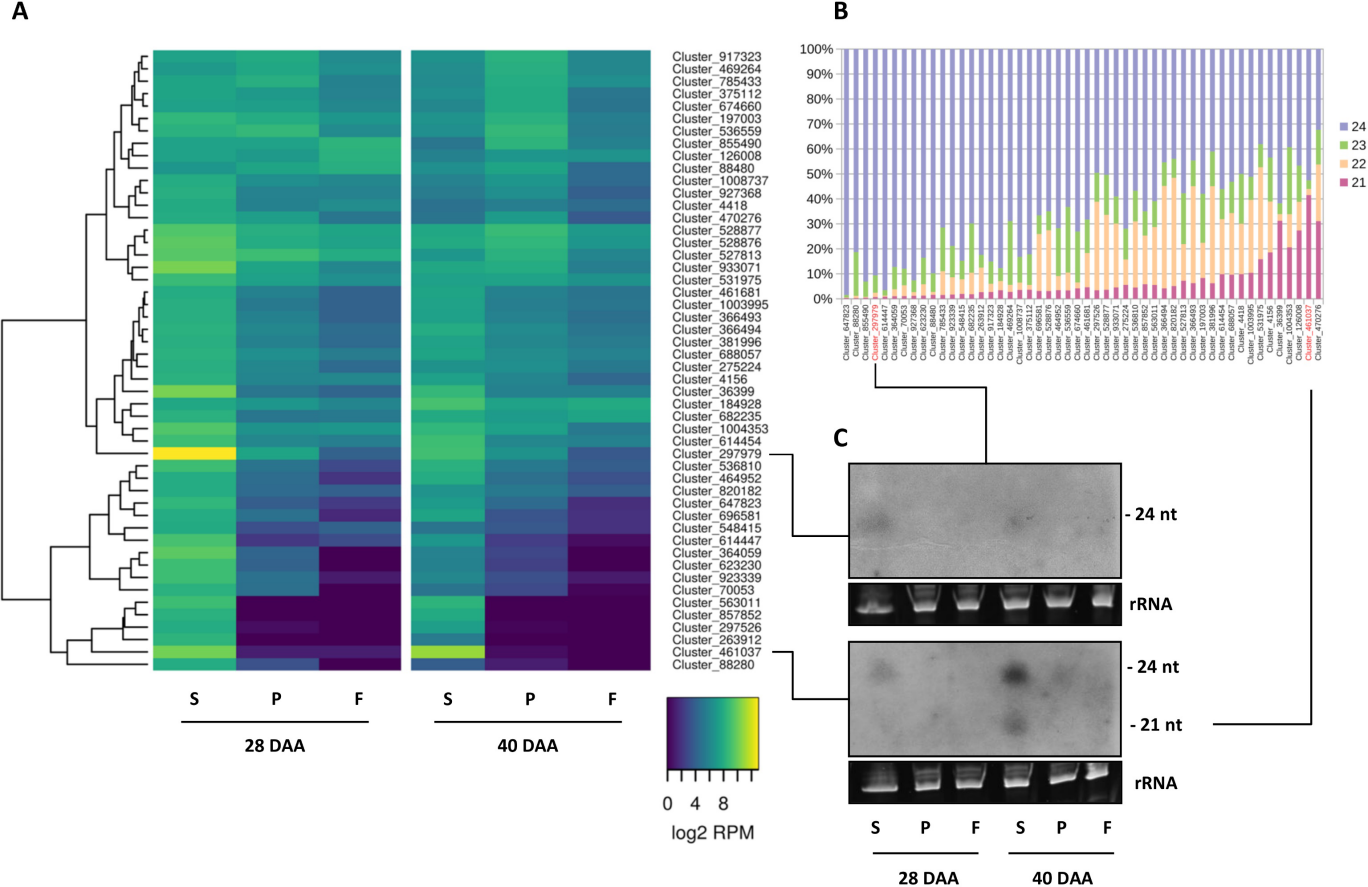
Expression pattern and sequence length composition of 24-nt-long hetsiRNA-producing clusters. Heat map showing (A) the log2 transformed expression levels (Read Per Million) and (B) sequence length composition of the 50 most abundant clusters, and (C) Northern blot analyses of the expression of Cluster 297979 and Cluster 461037.

The detailed analyses of the top 50 most abundant 24-nt hetsiRNA-producing clusters revealed tissue-specific and temporally highly regulated expression patterns (Fig 7A, Table oo in S2 File). Some siRNA-producing clusters expressed at a very high level in the seed at 28 DAA, drastically reduced at 40 DAA, and almost completely silent in other tissues.

To verify this remarkable tissue- and developmental stage-specific expression of the named clusters we investigated the expression of two representative clusters by sRNA Northern blot analyses (Fig 7C). Cluster_297979 is pepper-specific, covering approximately a 5 Kb-long intergenic region and expresses at a very high level. The locus is stranded but cannot be folded into a simple hairpin structure. A dominant 24-nt-long siRNA species is produced from the negative strand. Cluster_461037, approximately 1 Kb long, exhibits an opposite pattern of expression compared to Cluster_297979, showing enhanced expression at 40 DAA in the seed (Fig 7A). This locus was described earlier as a miRNA gene (*can-miR-n008*) [25], but in our dataset, it looks more like an inverted repeat-associated siRNA locus. The dominant 24-nt-long sequence contains one mismatch compared to the *can-miR-n008* mature sequence. Furthermore, no star sequence was found in any of the libraries. Under our experimental conditions, an abundant 21-nt-long siRNA species is also produced. In line with the NGS data, we detected the enhanced expression of both abundant sequence species in the seed at 28 DAA which declined at 40 DAA using DNA probe specific to the dominant siRNA species (Fig 7C). At this sensitivity level, in agreement with the NGS data, both probes detected no signals in the flesh and the placenta samples.

Altogether, these data reveal a dynamic spatiotemporal regulation of the expression of hetsiRNAs in the developing pepper fruit. These observations are in accordance with previous Arabidopsis results demonstrating the dynamic expression of Pol IV-dependent siRNAs during silique development [51]. It has been shown that the Pol IV-dependent siRNA accumulation is initiated in the maternal gametophyte and continues in the seed, predominantly in the developing endosperm. Our observation that siRNA clusters are activated in a developmental phase-dependent and tissue-specific manner suggests that hetsiRNAs are potent regulatory components of the pepper fruit development.

### Expression of phased siRNA and secondary siRNA-producing clusters

Phased secondary siRNAs are produced from Pol II-dependent transcripts that are cleaved by a predominantly 22-nt-long phase-initiating miRNA [52]. One exception is *TAS3*, which is cleaved by a 21-nt-long *miR390* by a different mechanism [53]. This cleavage then initiates the synthesis of a complementary RNA by an RNA-dependent RNA polymerase (RDR6 in Arabidopsis), resulting in a double-stranded intermediate that is diced by the DCL4 endonuclease at every 21-nt starting from the miRNA cleavage site. ShortStack identified 93, and 948 potential phased clusters of 21-nt and 24 nt, respectively, the 50 most abundant are shown in Figures H and I in S1 File (the associated data are in Tables pp and qq in S2 File). The majority of the 21-nt PHAS loci overlap with genes coding for leucine-rich repeat-containing receptors, which a play a role in pathogen resistance and fruit ripening as well [54]. They have already been described as phasiRNA-producing genes in other species. The phasing is usually initialized by miRNAs belonging to the *miR482*/*miR2118* family or by other phasiRNAs. Surprisingly, we could not identify *TAS* homolog genes by BLAST alignment of Arabidopsis or other known plant *TAS* sequences to the Zunla RNA sequences, possibly because only the functional tasiRNA sequences are conserved, not the full noncoding transcripts. We searched for short matching patterns to the known tasiRNA sequences [55] using Patman [36]. This way we managed to identify three possible *TAS3* homologue genes (*ARF2-like*, *ARF3*, *ARF4*), two *TAS4* homologous genes (Potassium transporter 5 and Aquaporin PIP2-1-like) with microhomology only to the conserved tasiRNA sequences, and two *TAS1-like* genes (coding for AT4G04775-like uncharacterized protein and ATP-dependent DNA helicase Q-like 5) that can only be found in apple and peach, according to the tasiRNA database [55]. We did not find these potential *TAS* loci to produce phased siRNAs, probably because their known phase initializing miRNAs are hardly expressed in all of our libraries. Beside the above mentioned large protein family, there are single phasiRNA-producing genes, which are more specific to certain plants. It is worth mentioning that a *DICER-LIKE PROTEIN 2-like* gene is also a phased siRNA producing locus under our experimental conditions, which was also found to be a PHAS locus in *Glycine max* and *Medicago truncatula* [8] [56]. Moreover, this locus was differentially expressed between 28 and 40 DAA, in all tissues. DCL2 is a member of the sRNA processing machinery that plays a role in endogenous and virus-derived siRNA production.

Some 24-nt PHAS loci have been described earlier [57, 58] and appear to be developmentally regulated. It is noteworthy that the most abundant 24-nt PHAS loci we identified are also developmental stage-specific, they are much more abundant in the seed sample at 28 DAA (Figure I in S1 File). According to the previous findings, these loci are associated with transposons, and their function is, similarly to the PIWI-associated siRNA in animals, to maintain genomic stability in reproductive tissues.

## Conclusion

In conclusion, our work provides comprehensive tissue-specific miRNA and siRNA expression landscape linked to expansion of pepper fruit. The separated analyses of fruit tissue components allowed us to dissect and list the sRNA components dominantly expressing in different tissue types in the expanding pepper fruit at two time points. Our analyses revealed several abundantly expressing tissue- and pepper-specific small regulatory RNA species implicating their regulatory role. Altogether, the presented data extend the potential perspectives of sRNA-mediated regulatory processes associated with the complex program of fruit development and provide fundamental data sets for further experiments.

## Supporting information

S1 File Supplementary Figures. Figures A-I.
(PDF)

S2 File Supplementary Tables. Tables A-Z and Tables aa-rr.
(XLSX)
