# Correction: Expansion of *Capsicum annum* fruit is linked to dynamic tissue-specific differential expression of miRNA and siRNA profiles

**DOI:** 10.1371/journal.pone.0203582

**Published:** 2018-08-30

**Authors:** Dénes Taller, Jeannette Bálint, Péter Gyula, Tibor Nagy, Endre Barta, Ivett Baksa, György Szittya, János Taller, Zoltán Havelda

The word “*annum*” is misspelled in the article title. The correct title is: Expansion of *Capsicum annuum* fruit is linked to dynamic tissue-specific differential expression of miRNA and siRNA profiles. The correct citation is: Taller D, Bálint J, Gyula P, Nagy T, Barta E, Baksa I, et al. (2018) Expansion of *Capsicum annuum* fruit is linked to dynamic tissue-specific differential expression of miRNA and siRNA profiles. PLoS ONE 13(7): e0200207. https://doi.org/10.1371/journal.pone.0200207
