# Supplementary material for: Expansion of Capsicum annum fruit is linked to dynamic tissue-specific differential expression of miRNA and siRNA profiles
Source: PLoS One. 2018 Jul 25;13(7):e0200207. doi: 10.1371/journal.pone.0200207 (PMC6059424; doi:10.1371/journal.pone.0200207)
Supplement: S1 File — Figures A-I. (PDF) [file pone.0200207.s001.pdf]

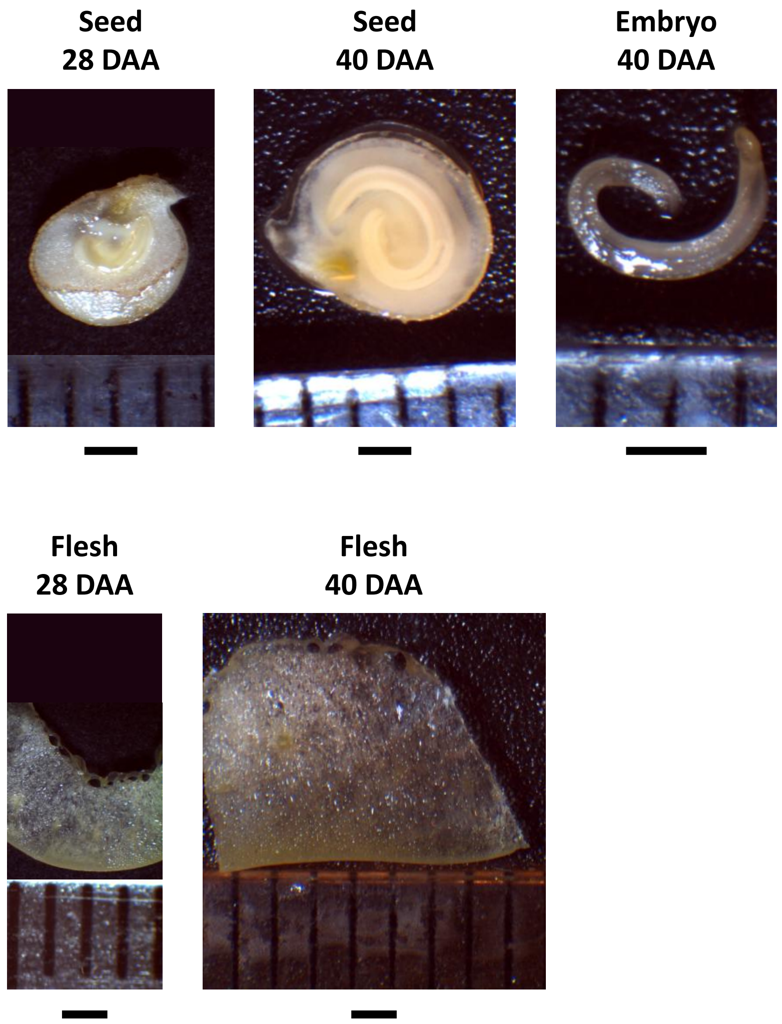

Figure A. Close-up view of tissues of the pepper fruit at 28 and 40 DAA.

At these stages the differentiation has already completed, only the increase in size can be observed. Bars represent 1 mm.

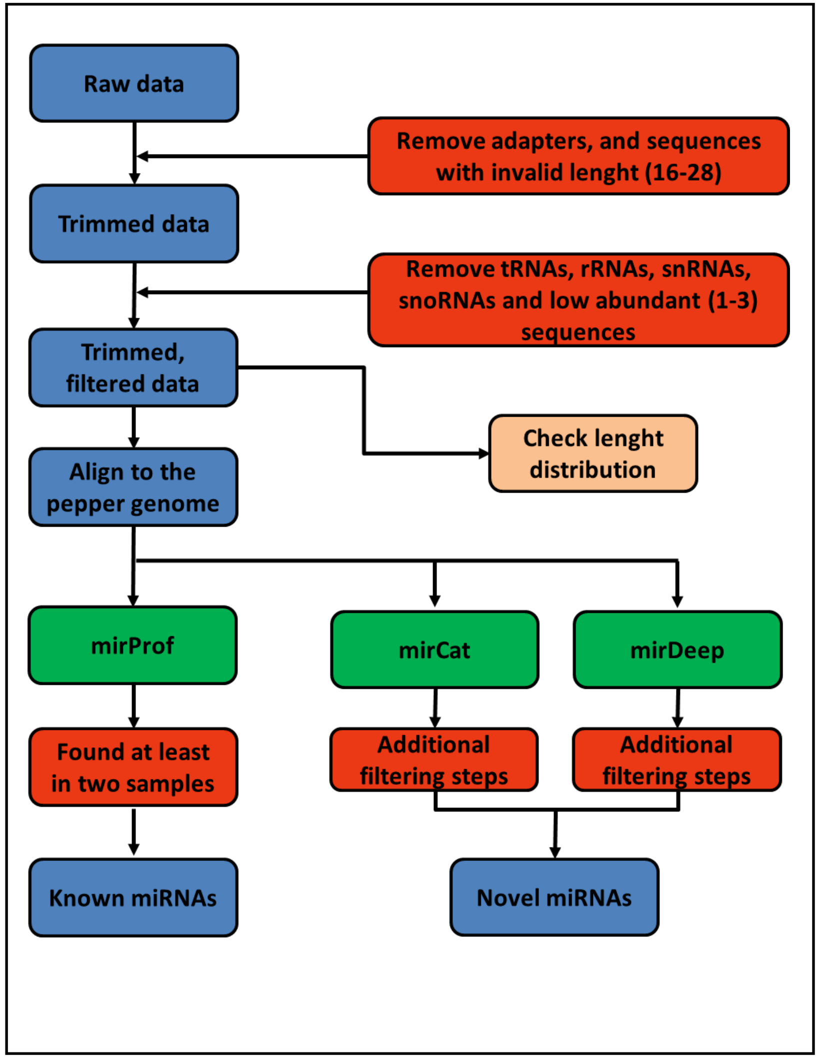

Figure B. Flowchart of the steps of new pepper specific miRNA prediction. Raw reads were processed using `cutadapt` to remove adapter sequences and filter out low quality reads. The filtered sequences were aligned to the Rfam database. Non-matching sequences were aligned to the pepper genome (CM334). Three different miRNA prediction tools were utilised. `mirProf` and `mirCat` are part of the `UEA sRNA Workbench`, while `mirDeep-P` is a modified version of `mirDeep` tailored for plant miRNA prediction. `mirProf` was used to align sequences to miRBase, a database of known miRNAs or a home-made database of known pepper-specific miRNAs. Sequences that did not match known miRNAs were used for novel miRNA identification. Only those potential miRNAs were accepted in which case the star sequence was found. The results of the two different tools were compared and only the common elements were retained.

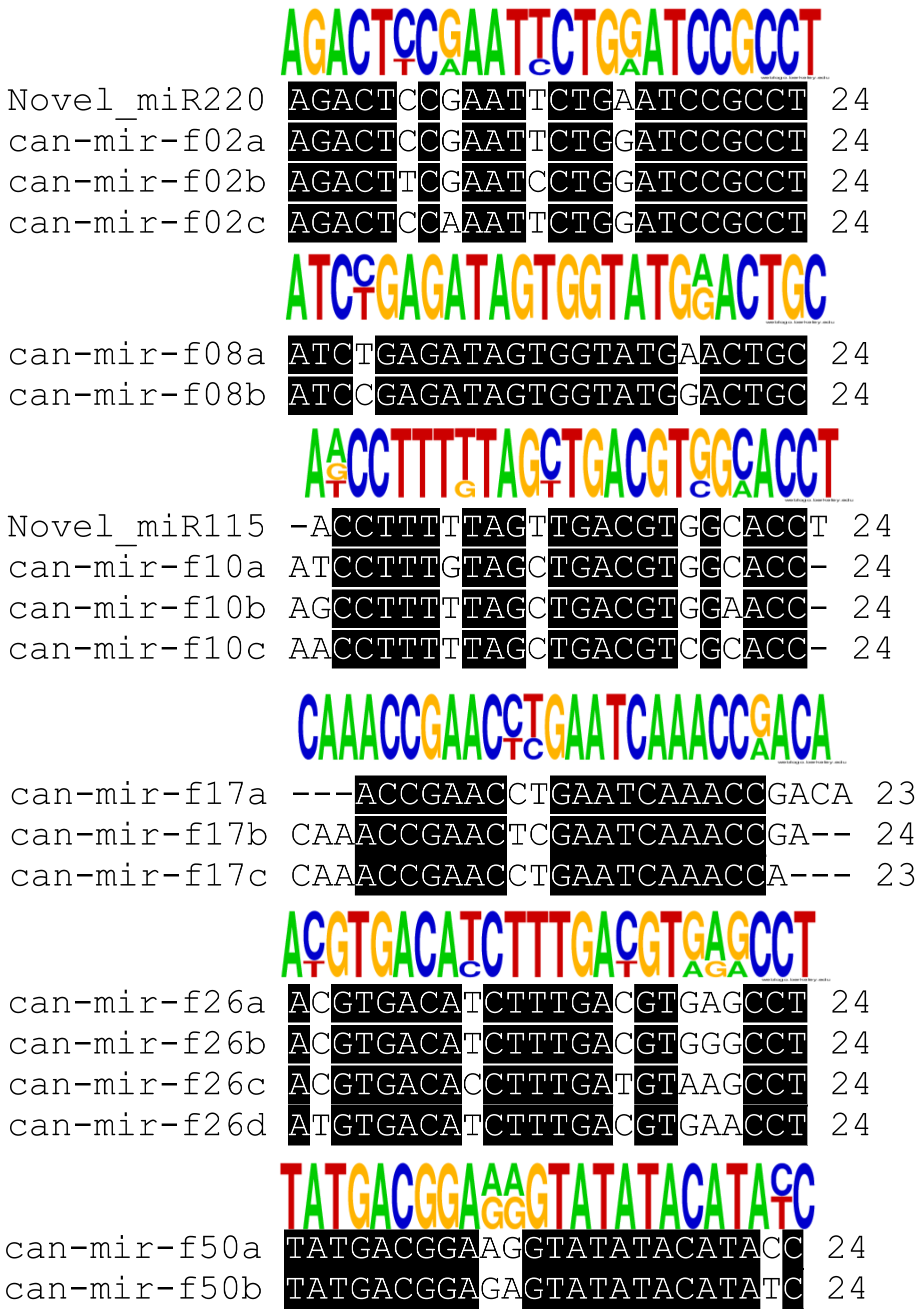

Figure C. Novel miRNA family members. The diagram shows all the new miRNA families. A consensus sequence logo is shown for each family, the sequence alignment of the clustered miRNAs are indicated below the logo.

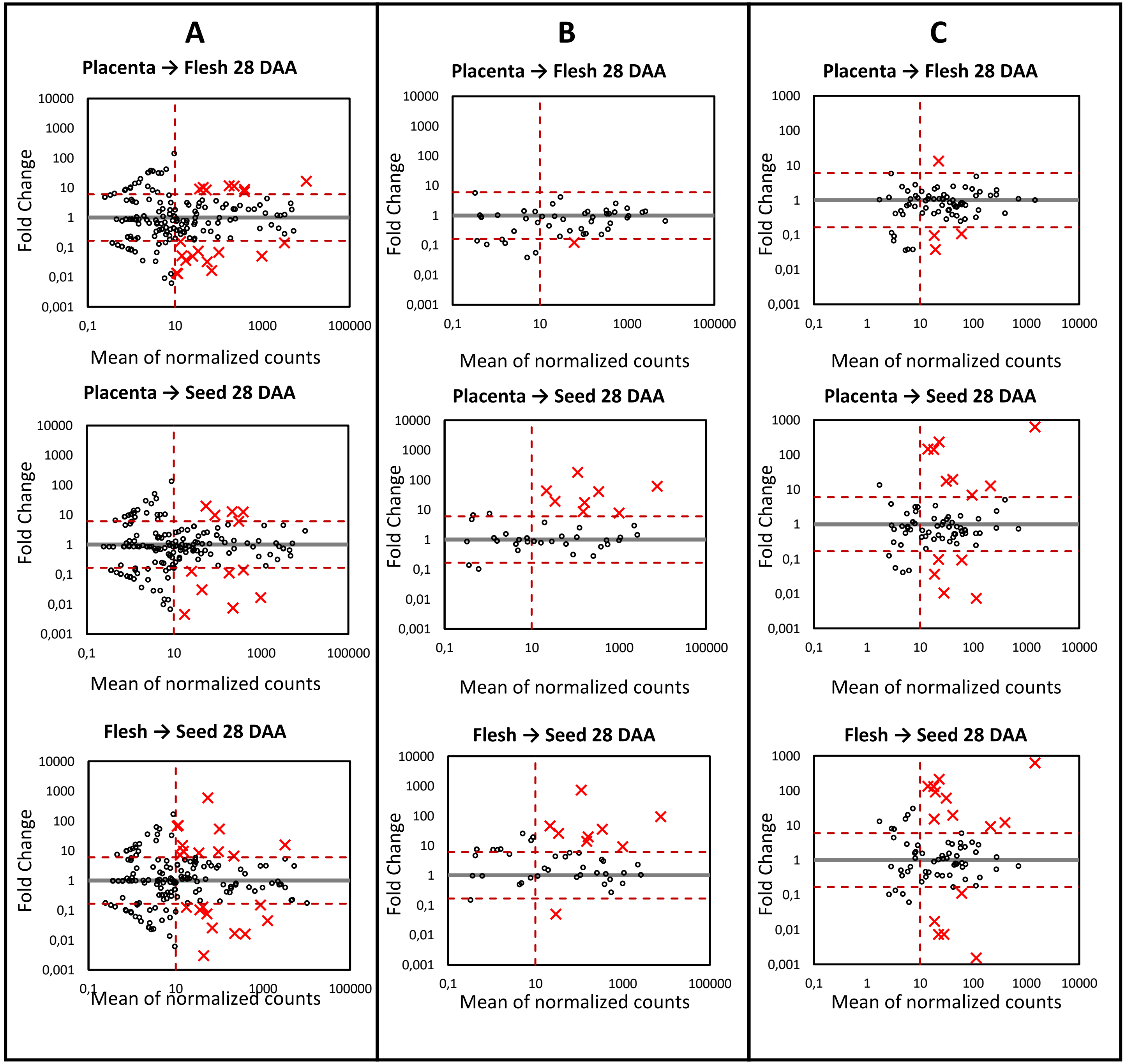

Figure D. Differential expression of conservative (A), known(B) and novel(C) miRNAs between different tissues in the 28 DAA stage vizualized on MA plots. DESeq2 was used for normalization and differential expression analysis. The X axis shows the basemean values, and Y shows the Fold change. Red crosses show the changing miRNAs, which are above the basemean limit (10) and shows an at least 6-fold change. We labeled these limits with red dashed lines. Black dots represent miRNAs that we did not consider changed.

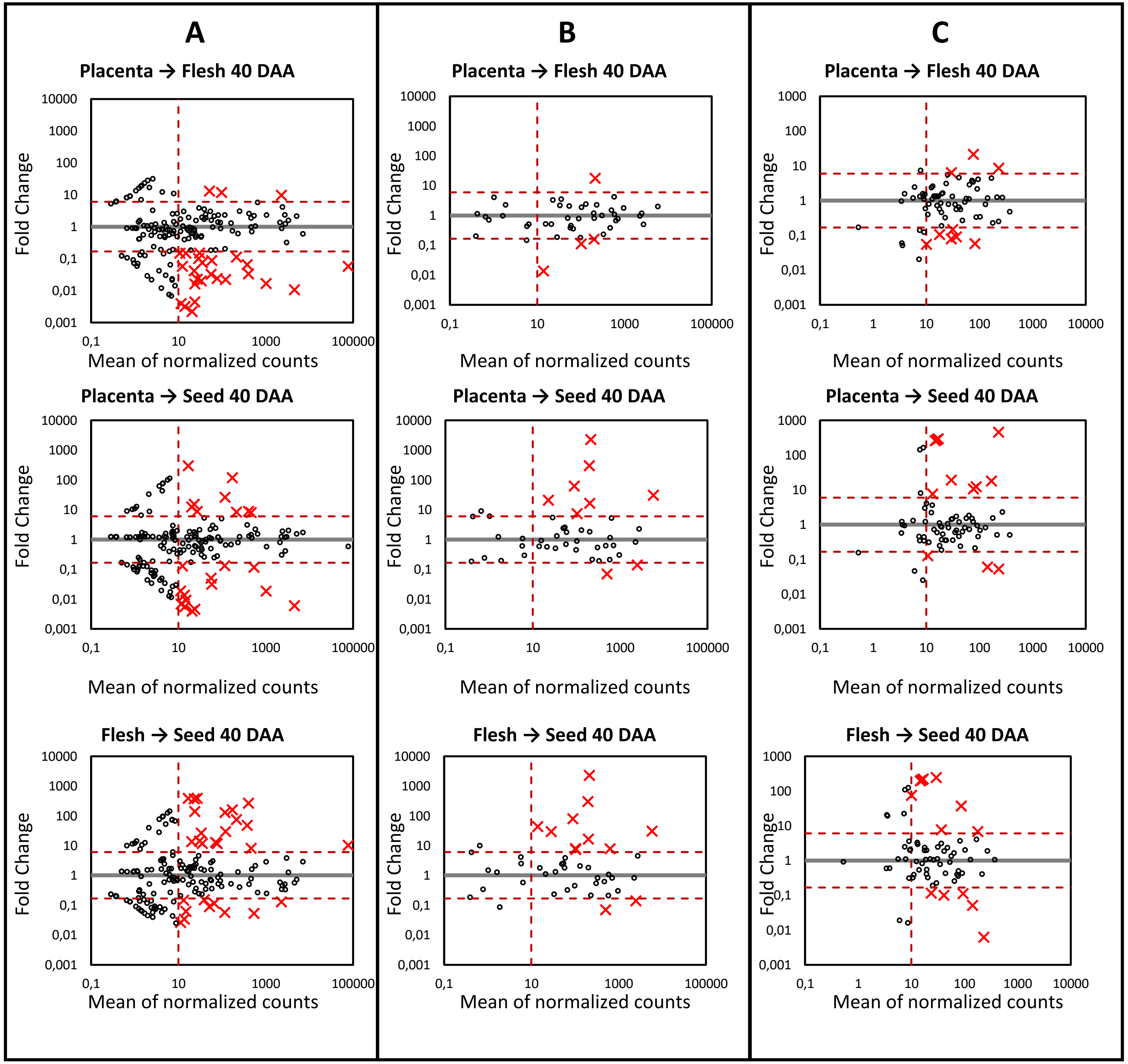

Figure E Differential expression of conservative (A), known(B) and novel(C) miRNAs between different tissues in the 40DAA stage vizualized on MA plots. DESeq2 was used for normalization and differential expression analysis. The X axis shows the basemean values, and Y shows the Fold change. Red crosses show the changing miRNAs, which are above the basemean limit (10) and shows an at least 6-fold change. We labeled these limits with red dashed lines. Black dots represent miRNAs that we did not consider changed.

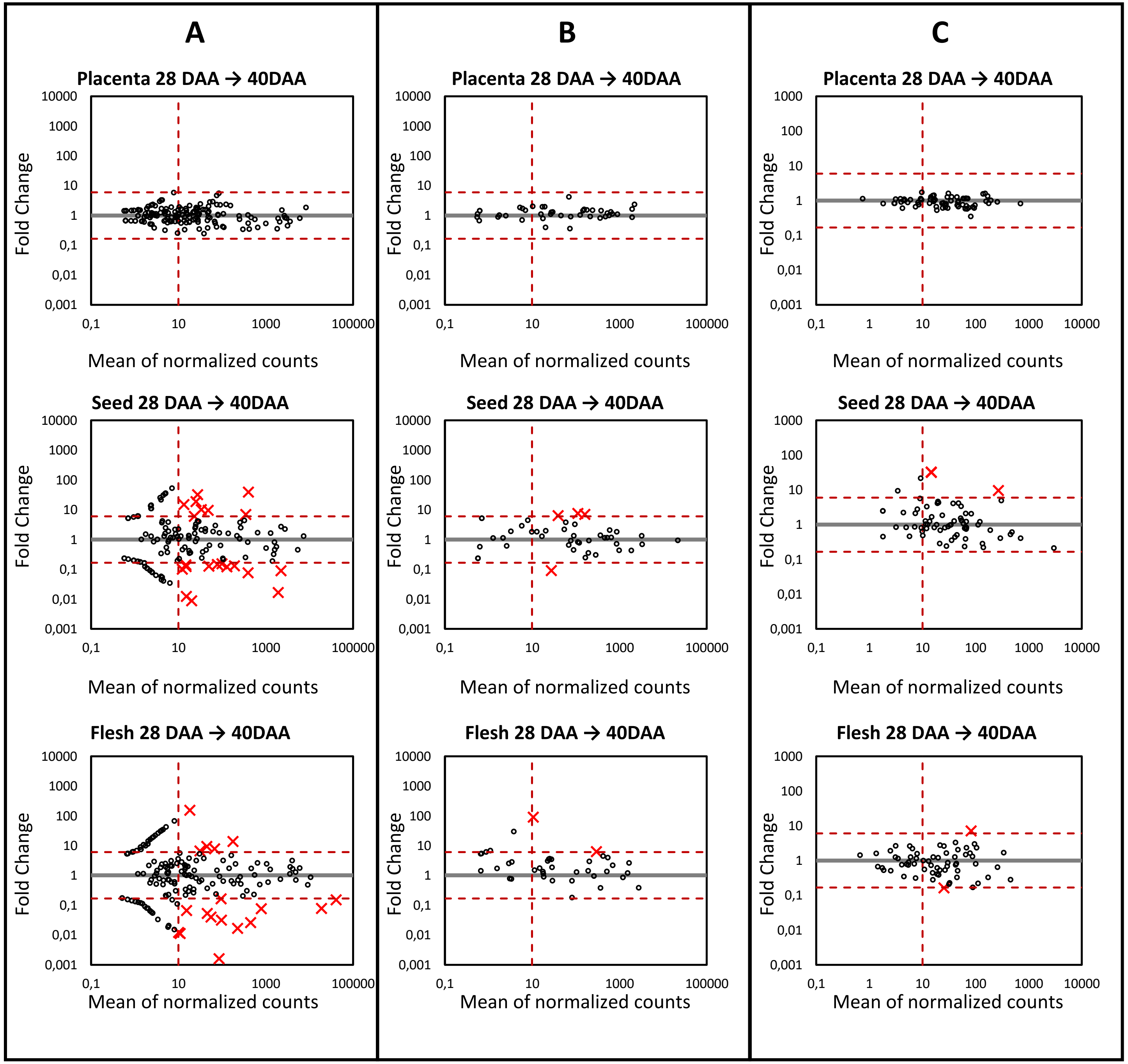

Figure F Differential expression of conservative (A), known(B) and novel(C) miRNAs between the two developmental stage vizualized on MA plots. DESeq2 was used for normalization and differential expression analysis. The X axis shows the basemean values, and Y shows the Fold change. Red crosses show the changing miRNAs, which are above the basemean limit (10) and shows an at least 6-fold change. We labeled these limits with red dashed lines. Black dots represent miRNAs that we did not consider changed.

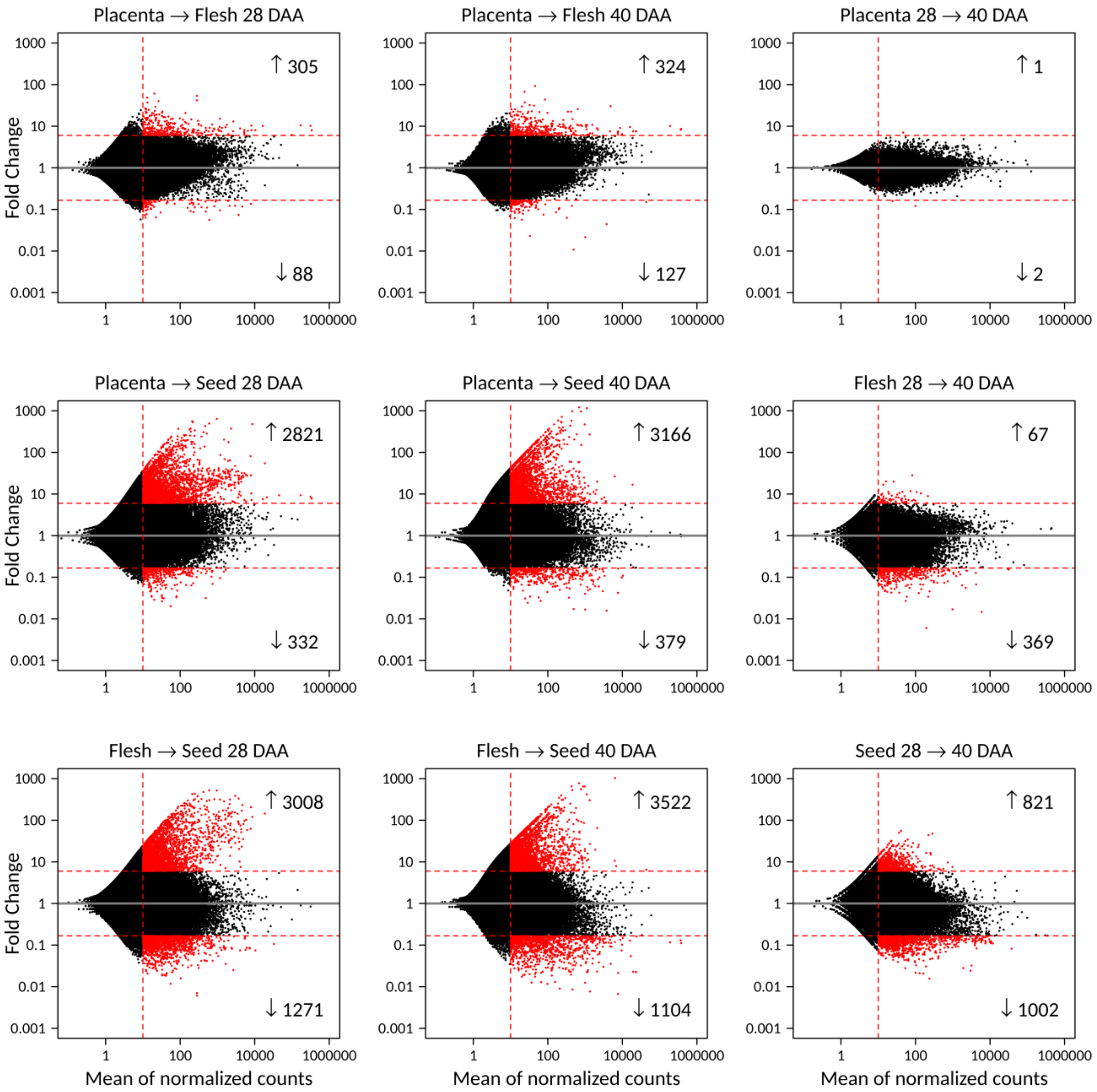

Figure G. Differential expression of sRNA clusters visualized on MA-plots. Normalization and differential expression analysis was performed with DESeq2. The X axis shows the basemean values, and Y shows the Fold change. Red crosses show the changing miRNAs, which are above the basemean limit (10) and shows an at least 6-fold change. We labeled these limits with red dashed lines. Black dots represent sRNA loci that we did not consider changed. The number of the up- and downregulated loci are shown in the corner.

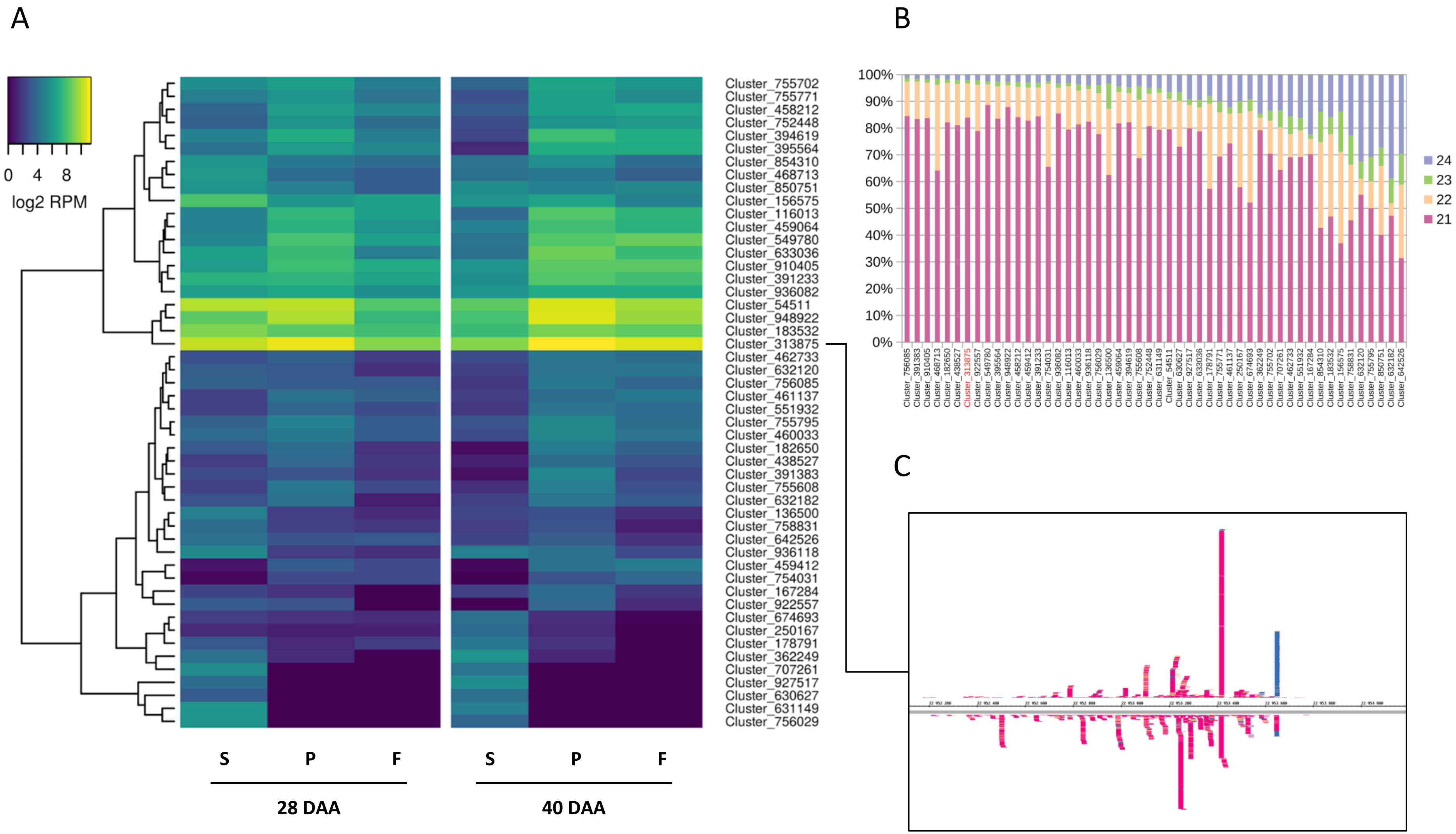

Figure H. Expression pattern and sequence length composition of 21-nt-long phased siRNA clusters. Heat map showing the log2 transformed expression levels (Read Per Million) of the 50 most abundant clusters (A). Sequence length composition of the 50 most abundant 21-nt long phasiRNA producing clusters (B). Genomic view of Cluster_313875 (C).

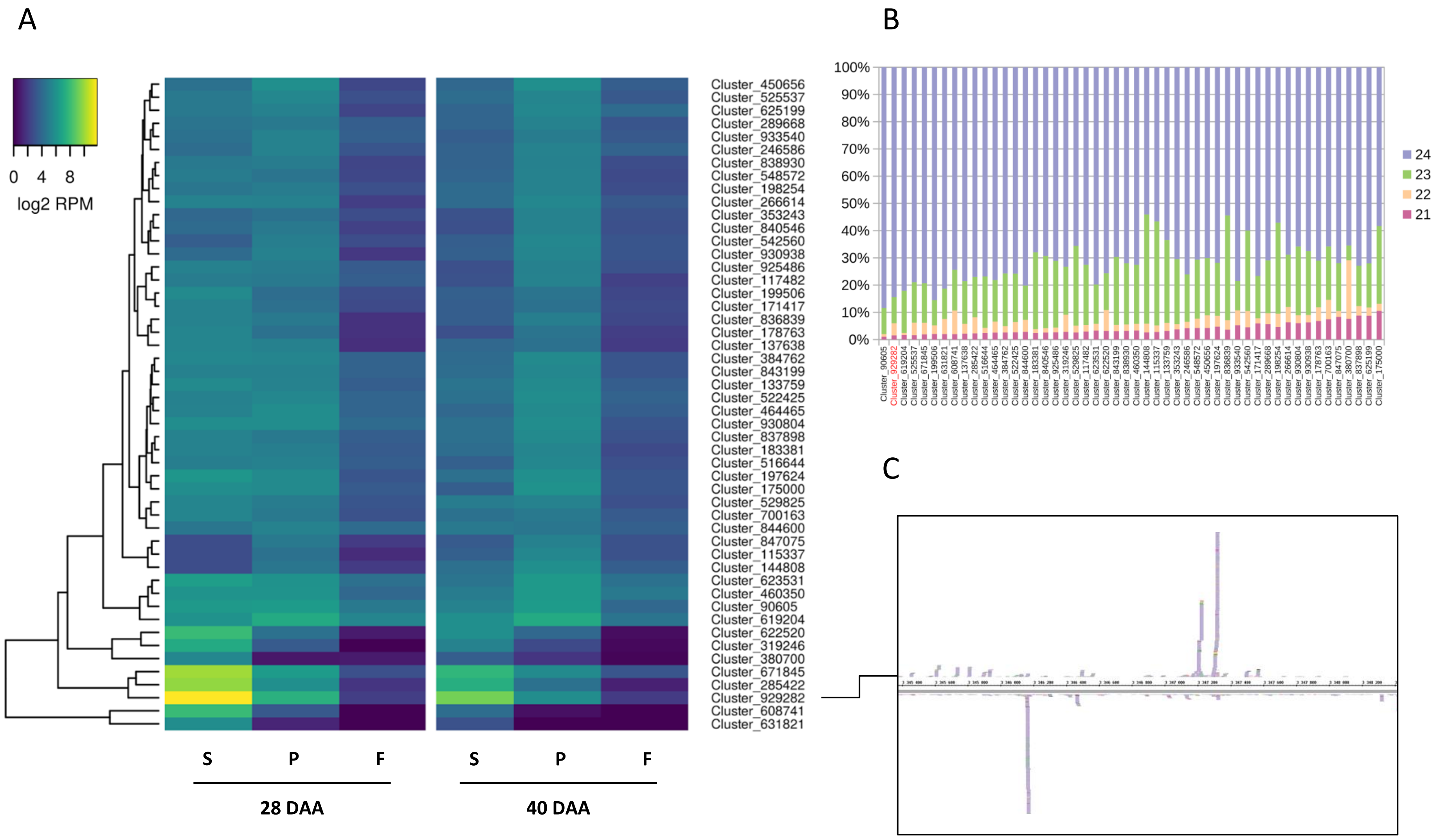

Figure I. Expression pattern and sequence length composition of 24-nt-long phased siRNA clusters. Heat map showing the log2 transformed expression levels (Read Per Million) of the 50 most abundant clusters (A). Sequence length composition of the 50 most abundant clusters (B). Genomic view of Cluster_929282 (C).
